# Interbrain Synchrony Mitigates Extremism Within Echo Chambers

**DOI:** 10.1111/nyas.70083

**Published:** 2025-09-12

**Authors:** Aial Sobeh, Tomer Marcos Vakrat, Simone Shamay‐Tsoory

**Affiliations:** ^1^ Social and Affective Neuroscience Lab, Department of Psychology University of Haifa Haifa Israel; ^2^ The Integrated Brain and Behavior Research Center (IBBRC) Haifa Israel

**Keywords:** echo chambers, extremism, fNIRS, group, interbrain synchrony, morality

## Abstract

People tend to engage with content that aligns with their pre‐existing attitudes, forming echo chambers that reinforce biases and may amplify extremism. Here, we investigate whether discussions within homogeneous groups drive attitudinal extremity and whether interbrain synchronized activity between the executive control brain regions of group members can moderate this relationship between homogeneity and increased extremity. One hundred and eighty‐eight participants were randomly divided into groups of four individuals. They then engaged in a moral judgment task in which they privately rated and then discussed the appropriateness of actions taken to resolve moral dilemmas, while their brain activity was scanned using functional near‐infrared spectroscopy (fNIRS). Group homogeneity was evaluated using participants' pre‐discussion private ratings, while extremism was measured based on how extreme their post‐discussion private ratings were compared to their pre‐discussion ratings. Our results show that discussions within homogeneous, compared to heterogeneous groups, led to adopting more extreme views. Critically, we found that higher interbrain synchrony between group members’ dorsolateral prefrontal cortex (DLPFC) during discussions diminishes this effect of homogeneity on extremism. We propose that interbrain synchrony in the DLPFC can counter harmful interpersonal mechanisms that take place within an echo chamber environment.

## Graphical abstract

People engage with content that aligns with their pre‐existing attitudes, forming echo chambers and amplifying extremism. Participants engaged in a moral judgment task where they privately rated and discussed the appropriateness of actions taken to resolve moral dilemmas. Brain activity was scanned using fNIRS. Discussions within homogeneous, compared to heterogeneous groups, led to adopting more extreme views. Higher interbrain synchrony between group members’ DLPFC during discussions diminished the amplifying effect of homogeneity on extremism.

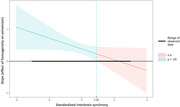

## 1 Introduction

Ideological extremism can occur when the beliefs or attitudes of individual group members become more extreme following exposure to social information [1, 2]. Increased extremism can fuel polarization and can hinder communication and cooperation, exacerbate conflict, and heighten hostility between opposing groups as their ideological overlap shrinks [3]. Given its potential risks, it is important to identify the processes fueling extremism and characterize the mechanisms needed to reduce it. Extensive research has demonstrated that, specifically within ideologically homogenous groups, members tend to gravitate toward more extreme views following group discussions [2]. The increasing extremity of views within homogenous groups was termed the echo chamber effect and is thought to be a catalyst for the rise in polarization [2, 4]. Research on motivated information processing suggests that this effect arises because members of homogenous groups primarily encounter information and arguments reinforcing their pre‐existing positions [5, 6, 7]. Group members in turn are less inclined to critically evaluate these shared beliefs, as they are motivated by a desire for confirmation and group cohesion [5, 6, 7]. This interaction between selective exposure and the lack of critical evaluation fosters a confirmatory rather than a critical mindset [8], leading to overconfidence in the group's existing views, and to greater susceptibility to extreme positions especially when voiced by dominant ingroup figures. We thus propose that a neural mechanism supporting a critical and flexible group mindset may help protect against extremism.

Recent behavioral research suggests that the manner in which information is communicated and evaluated during group discussions plays a key role in determining the likelihood of extreme views emerging [9, 10]. Communicating and evaluating beliefs through discussions is an interactive process that involves two or more communicating individuals attempting to influence, understand, and learn from one another [11]. To investigate when and how this process shapes behavior, recent research has adopted a second‐person neuroscience approach, examining the neural mechanisms underlying communication and social interaction [12, 13]. Studies using hyperscanning paradigms—which involve measuring brain activity from multiple individuals during social interactions—consistently demonstrate that the neural activity of interacting individuals becomes synchronized during shared tasks [14, 15]. This phenomenon, termed interbrain synchrony, has been associated with interpersonal cooperation, coordination, and communication success [14, 16].

While interbrain synchrony is observed between many brain regions across brains, with different patterns predicting different behavioral outcomes, interbrain synchrony in the dorsolateral prefrontal cortex (DLPFC) has been specifically associated with group creativity and collaborative behavior [17, 18], suggesting that it plays a role in fostering cognitive flexibility and cooperative mindsets [14, 16]. The DLPFC is a core region in the executive control network (ECN), a brain network comprising the frontal and parietal cortices, and supporting effortful and flexible thinking [19, 20]. Research involving both healthy individuals and patients with DLPFC damage underscores this region's critical role in facilitating adaptive thought processes [21] and inhibiting automatic or habitual responses [22]. Individuals with lesions in the DLPFC also exhibit increased dogmatism and religious fundamentalism, linking impairments in this region to rigid and extreme belief systems [23]. These findings highlight the DLPFC's essential function in tasks requiring mental flexibility—the ability to adjust behaviors and beliefs in response to changing environmental demands.

Building on previous findings, we hypothesized that interbrain synchrony within the DLPFC could help mitigate the echo chamber effect by diminishing the emergent extremism following discussions within homogeneous groups.

**FIGURE 1:**
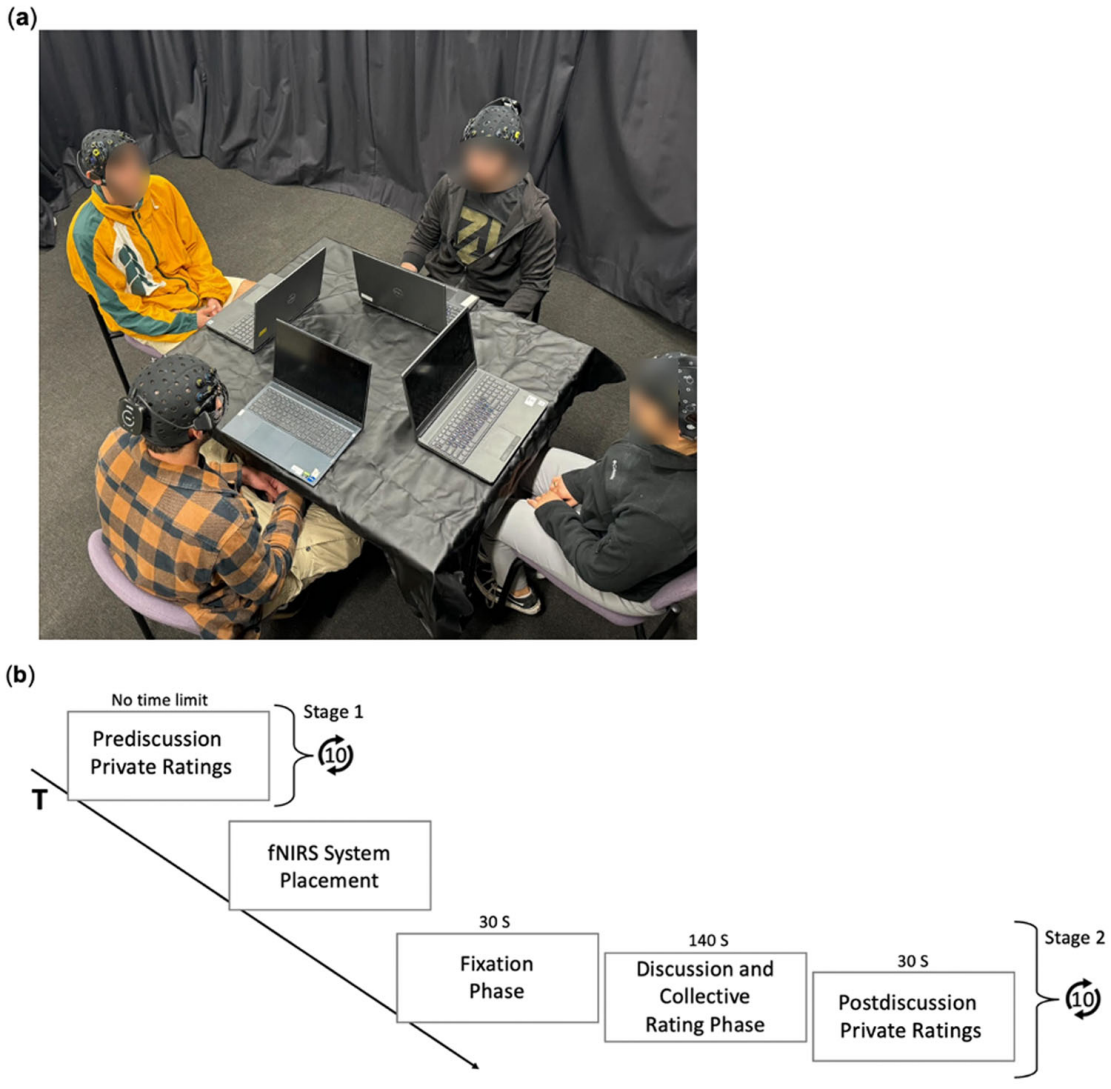
Seating arrangement and moral judgment paradigm timeline. (a) A picture depicting participants’ seating arrangements and (b) the moral judgment paradigm timeline.

To evaluate our hypothesis, we used functional near‐infrared spectroscopy (fNIRS) to scan participants, divided into groups of four, as they engaged in discussions of moral dilemmas. The task involved presenting participants with a series of moral dilemmas resembling the trolley problem and asking them to rate the morality of controversial actions taken to resolve each of these dilemmas (Figure 1). Group homogeneity was assessed based on the similarity of participants’ pre‐discussion ratings, while extremism was measured by the extent to which participants’ post‐discussion ratings became more extreme compared to their pre‐discussion ratings. We hypothesized that deliberation in homogeneous groups, compared to heterogeneous groups, would result in greater extremism, thereby replicating the echo chamber effect in a controlled lab setting. Furthermore, we predicted that higher interbrain synchrony among group members in the DLPFC would moderate this effect by weakening the association between group homogeneity and extremism.

## 2 Methods

### 2.1 Participants

A total of 220 individuals were initially recruited and randomly assigned into 55 groups, with four participants each. The number of groups was determined based on power analysis with an assumed moderate effect size, *α* = 0.05, and power of 0.80 [24]. Due to data acquisition issues, eight groups were excluded from the analysis, resulting in a final sample of 47 groups (188 participants; 133 female). The data acquisition problems were primarily caused by an unstable bluetooth connection between the neuroimaging device and the data acquisition software during the brain activity recording sessions. These connection issues led to multiple disconnections and an unsystematic loss of neuroimaging samples from participants in the excluded groups, which precluded their inclusion in the final analysis.

Participants were recruited individually through advertisements throughout the University of Haifa campus or via social media, then randomly assigned to groups. This experiment formed part of a larger lab study on group dynamics and social conformity. The same set of raw data gathered from these groups were analyzed in another study that examined social conformity; while the raw neural activity and ratings data were similar in the two studies, the extracted interbrain synchrony measures and the behavioral measures reported here were specific to the current study. Exclusion criteria included left‐handedness (due to differences in brain lateralization that may introduce variability in the data), reading difficulties, and a history of neurological or psychiatric disorders. Members of each group shared a native language (Hebrew or Arabic), and the study was conducted in the participants' native language to enhance comprehension and expression (13 groups in Hebrew, 34 in Arabic).

This study was reviewed and approved by the Institutional Review Board (IRB) of the University of Haifa (Approval Number: 351/19). All procedures performed in this research adhered to the ethical standards of the University of Haifa Ethics Committee and the principles of the Declaration of Helsinki. Participants provided informed consent before participation, and their confidentiality and privacy were ensured throughout the study.

### 2.2 Procedure

#### 2.2.1 Pretask Arrangements

Participants were randomly seated facing each other, each in front of an individual computer screen (see Figure 1a). Participants were first asked to sign a consent form and complete a demographic questionnaire and a questionnaire assessing their levels of acquaintance with each of the other group members, on a four‐point semantic differential scale ranging from “complete strangers” to “best friends/romantic partners.” No group included two or more best friends or romantic partners.

#### 2.2.2 Moral Judgment Paradigm

The task consisted of two stages (Figure 1b). In stage 1, participants read a series of written descriptions of 10 moral dilemmas resembling the classic trolley problem. Each scenario presented a different situation where the protagonist had to choose between utilitarian (outcome‐focused) and deontological (principle‐based) options. At the end of each scenario, a utilitarian decision was suggested as a resolution. Participants rated the moral appropriateness of this decision using an eight‐point semantic differential scale, with labels and numbers from 1 (completely morally inappropriate) to 8 (completely morally appropriate), without a neutral midpoint (see Figure S1 for an example of one dilemma and the scale as they were presented to the participants). The dilemmas appeared sequentially on the computer screen, and participants could proceed from one screen to the next only after rating the current dilemma. After stage 1, fNIRS devices (detailed in Section 2.4.1) were placed on participants’ heads, allowing us to measure their brain activity during stage 2 of the experiment.

In stage 2 participants encountered the same 10 dilemmas in 10 separate blocks that consisted of three phases. Each block started with a fixation phase where participants viewed a fixation cross at the center of their computer screen for 30 s. Participants were instructed to remain silent, motionless, and focused during the fixation presentation. Following the fixation phase, a dilemma appeared on all four screens of the four members of the group simultaneously. Participants then engaged in a group discussion phase, aimed at reaching a consensus (forced‐choice) on the moral rating of the dilemma, with 140 s allowed for deliberation and choice. Following the group discussion phase, the same dilemma reappeared on all screens, and then (at the post‐discussion private ratings phase) participants were asked to provide their own private rating within 30 s.

Our design ensured that each participant rated each of the 10 dilemmas three times: first privately pre‐discussion, then collectively through discussion, and then again privately post‐discussion (see Figure 1b).

### 2.3 Behavioral Measures

#### 2.3.1 Group Homogeneity

Homogeneous groups are composed of individuals that share the same moral inclination. In this study, participants were first classified as utilitarian or deontological with respect to each dilemma based on whether they rated the proposed utilitarian resolution for that dilemma as morally acceptable (ratings from 5 to 8; utilitarian), or as morally unacceptable (ratings from 1 to 4; deontological) in their pre‐discussion ratings. Then, a group was classified as homogeneous with respect to each given dilemma if all participants shared the same classification as utilitarian or as deontological. As a result, we obtained 10 observations for each group, one for each dilemma, with each one classified as homogeneous (dummy‐coded as 1) or heterogeneous (dummy‐coded as 0).

#### 2.3.2 Extremism

Extremism is defined here as a shift toward more extreme positions post‐discussion. Accordingly, we operationalized extremism at the individual level as the extent to which the participant's post‐discussion private rating for a given dilemma was closer to either extreme of the moral appropriateness scale (rating of one or eight) compared to their pre‐discussion private rating. For example, if a pre‐discussion rating was “3,” and a post‐discussion rating was “2,” then the post‐discussion rating would be one point closer to the nearest extreme (which is in this case “1”) compared to the pre‐discussion rating, and therefore the extremism score would equal 1. After computing the extremism scores for the four group members in each dilemma, we averaged the four scores to get a group‐level value of extremism, obtaining 10 observations for each group, one per dilemma.

#### 2.3.3 Homogeneity of Voiced Opinions

Participants engaged in group discussions about trolley moral dilemmas, and their conversations were recorded using smartphone audio recording devices (with each discussion recorded separately). The recordings were subsequently analyzed and coded by two independent coders. Each conversation was dummy‐coded as a “1” if only one side of moral reasoning (either deontological or utilitarian) was expressed, or as “0” if arguments in support of both sides of the moral debate were expressed. This allowed us to extract a dichotomous measure of homogeneity of voiced opinions, with one observation per discussion (10 per group). Inter‐rater reliability was 0.89 (agreement on 89% of cases). In instances where the two coders disagreed on the coding of a recording, they listened to the conversation together and resolved the discrepancy through consensus. For four out of the 47 groups in our study, audio recordings were not available, resulting in missing data for those groups with respect to this measure.

### 2.4 Neural Data Processing and Measures

#### 2.4.1 Neural Data Acquisition

**FIGURE 2:**
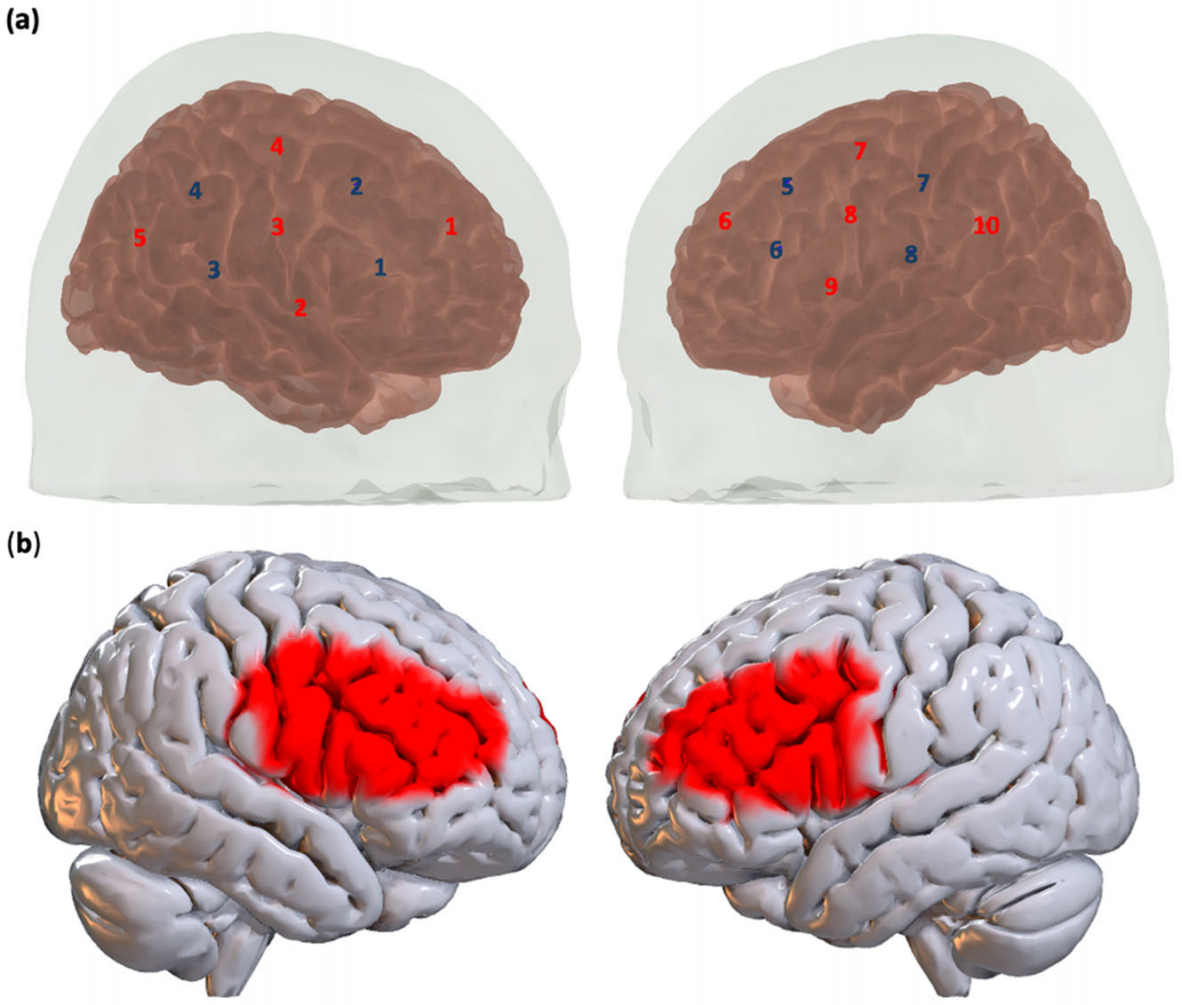
Channel Montage. (a) Illustration of the Brite24 fNIRS system channels montage using AtlasViewer MATLAB application [60]. Red numbers represent sources and blue numbers represent detectors. (b) Illustration of cortical surface covered by our montage (in red).

We utilized the Brite24 fNIRS system from Artinis Medical Systems (Elst, The Netherlands) to track fluctuations in cortical oxygenated (O2Hb) and deoxygenated hemoglobin (HHb) concentrations. This system uses dual‐wavelength near‐infrared light at 760 and 850 nm. Data were acquired at a sampling rate of 50 Hz. The system comprises a flexible probe headset with 18 optodes (10 sources and eight detectors), forming a total of 24 channels—12 on each hemisphere. The headset was placed on the participants' heads following the international 10–20 system, partially covering the bilateral prefrontal cortex (for channel montage, refer to Figure 2).

#### 2.4.2 Implemented Neural Data Preprocessing

Data preprocessing was performed using the HOMER3 MATLAB package [25], following five steps: (I) converting light intensity readings to optical density; (II) detecting and adjusting for motion artifacts via a targeted principal component analysis (PCA) approach [26]; (III) applying a bandpass filter with cutoff frequencies from 0 to 1 Hz to eliminate high‐frequency noise unrelated to brain activity [27]; (IV) converting optical density data into O2Hb and HHb concentration values—proxies for cerebral blood flow and neural activity—using the modified Beer–Lambert law [28]; (V) removing channels where O2Hb and HHb showed a positive correlation exceeding 0.5, as O2Hb and HHb typically demonstrate a strong negative correlation, and thus, high positive correlation characterizes noisy channels [29].

Channels were then clustered into 10 regions of interest (ROIs), and the preprocessed time series of channels within each ROI were averaged to produce a single time series per region. The grouping into ROIs was guided by the estimated Brodmann area (BA) below each channel's location. We estimated the BA by linking the digitized Montreal Neurological Institute (MNI) coordinates of each channel with its position according to the 10–20 system. The 10 ROIs included the following: bilateral Premotor Cortex (PMC), bilateral opercular part of the Inferior Frontal Gyrus (IFG's pars opercularis; BA44), bilateral triangular part of the IFG (pars triangularis; BA45), the bilateral DLPFC's BA46, and the bilateral DLPFC's BA9. Our selection of ROI's was guided by theoretical relevance and the technical constraints of fNIRS. We focused on the DLPFC due to its central role in executive control, cognitive flexibility, and deliberative decision‐making. We also included the IFG and PMC for their roles in mirroring and interpersonal synchrony [6]. Our 24‐channel montage does not offer full cortical coverage. We therefore prioritized regions that could be measured and were theoretically aligned with our aims.

#### 2.4.3 Wavelet Transform Coherence

To evaluate interbrain synchrony, we used the Wavelet Transform Coherence (WTC) technique, which quantifies coherence between two time series across specified time and frequency domains [30]. Coherence values—reflecting synchrony—range from 0, indicating no coherence, to 1, indicating full coherence. WTC was applied to O2Hb neural time series from each of the 10 pairs of corresponding ROIs across participants, resulting in 10 coherence matrices for each dyad of participants in any group. Coherence values were calculated for the frequency range from 0.16 Hz (6‐s period, aligning with the typical delay in the hemodynamic response [31]) down to 0.015 Hz (66‐s period). This frequency band was selected to avoid interference from respiratory (∼0.2–0.3 Hz) and cardiac (∼1–2 Hz) rhythms, and to capture the frequency range identified as optimal for wavelet coherence in fNIRS hyperscanning studies [32]. We implemented WTC using the wavelet coherence package in MATLAB [30].

#### 2.4.4 Group‐Level Interbrain Synchrony

Using the O2Hb coherence matrices obtained for each dyad of participants within a group (each group is made up of six unique dyads), we calculated an interbrain synchrony measure for each pair of ROIs (within each dyad) across the 10 deliberation and fixation phases. Specifically, coherence was averaged over both the designated frequency range and the duration of each phase. This process generated 200 interbrain synchrony observations per dyad—one for each ROI pair during each of the 10 deliberation phases and the 10 fixation phases (10 ROI pairs × 10 deliberation phases + 10 ROI pairs × 10 fixation phases). To derive a group‐level interbrain synchrony score, we averaged these dyadic synchrony values across all dyads within the group, resulting in 200 group‐level observations for each group.

#### 2.4.5 Pseudo Groups

To eliminate the possibility that interbrain synchrony is solely due to participants performing the same task simultaneously rather than from the interaction itself, we compared the levels of interbrain synchrony in real groups to pseudo groups. Pseudo groups were formed by randomly assigning participants to different groups. We generated 47 pseudo groups, ensuring that, like real groups, all members of a pseudo group shared the same native language. The shuffling process was conducted within these language groups, and no pair of participants appeared in more than one pseudo group. After generating pseudo groups, we calculated interbrain synchrony between members of those groups, allowing us to compare interbrain synchrony between real groups and pseudo groups.

### 2.5 Statistical Analysis

To examine the relationship between group type (real vs. pseudo) and interbrain synchrony, we employed hierarchical linear modeling (HLM) using the *lme4* package in R [33, 34]. HLM allowed us to account for the nested structure of the data, with participants nested within groups and responses nested within discussion items (i.e., the specific moral dilemmas discussed). When applied, group and item were included as random intercepts to account for potential clustering effects.

For moderation analyses, we constructed separate models for each ROI, testing whether interbrain synchrony moderated the relationship between group homogeneity (dummy‐coded) and post‐discussion extremism. Each model included homogeneity, standardized interbrain synchrony, and their interaction as fixed effects, with intercepts varying by group and dilemma. Model fitting was conducted using restricted maximum likelihood estimation.

To address the risk of Type I error due to multiple comparisons across ROIs, we applied Bonferroni correction for all sets of ROI‐level analyses, including those reported in Sections 3.3 and 3.4. The only exception was the Johnson–Neyman analysis used to identify regions of significance in the moderation effect. Because this technique involves testing across a continuous range of moderator values, we applied a false discovery rate (FDR) correction at a significance level of 0.05, consistent with standard practice for continuous interaction models.

## 3 Results

### 3.1 Homogeneity in Pre‐discussion Ratings Predicts Echo Chamber Environment

**FIGURE 3:**
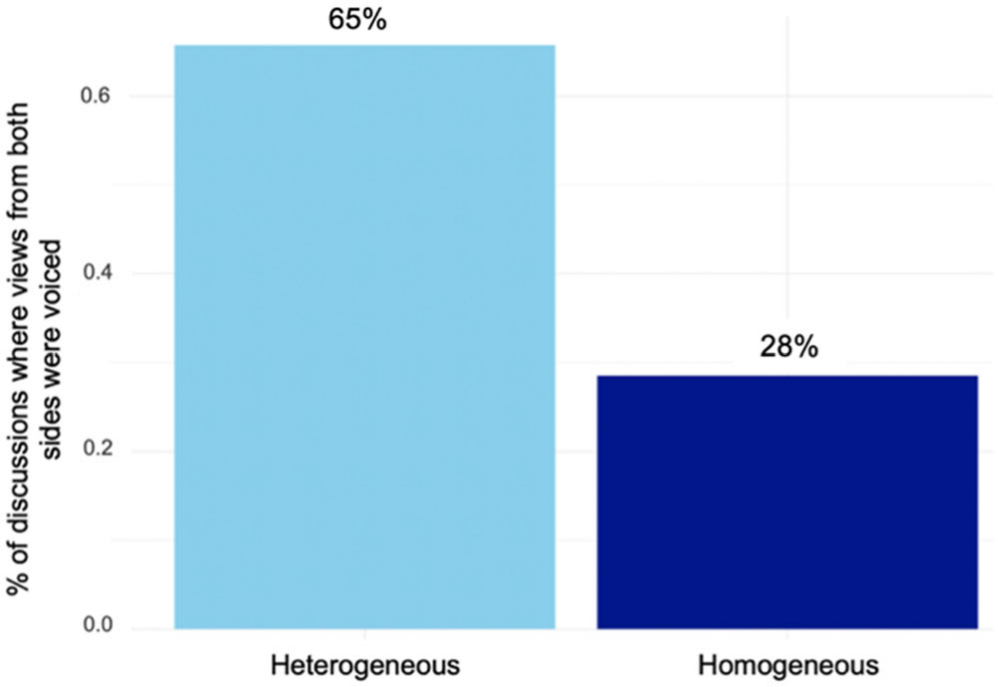
Homogeneity in pre‐discussion ratings predicts homogeneity in voiced opinions. Percentage of discussions in which views from both sides of the moral scale were raised as a function of whether the pre‐discussion ratings were homogenous or heterogeneous.

Pre‐discussion ratings were heterogeneous in 76.8% (*n* = 324) of the 422 discussions, and homogeneous in 23.2% (*n* = 98). Figure 3 illustrates the percentage of discussions in which views from both sides of the moral scale were voiced as a function of whether the initial ratings were homogeneous or heterogeneous. As shown, the percentage of discussions in which views from both sides of the moral scale were voiced is higher when pre‐discussion ratings were heterogeneous (65%) compared to when they were homogeneous (28%).

A chi‐square test of independence was conducted to examine this relationship between homogeneity in prediscussion ratings and the presence of views from both sides of the moral scale. The test revealed a significant association, (*χ* ^2^ (1, 422) = 40.934, *p* < 0.001), with Cramer's *V* indicating a large effect size (Cramer's *V* = 0.317). This strong association suggests that homogeneity in pre‐discussion private views promotes an echo chamber‐like environment during the discussion, where views from only one perspective are voiced.

Interestingly, we observed heterogeneity in voiced views during the discussion only in two‐thirds (65%) of discussions in which pre‐discussion views were heterogeneous. This suggests that in about one‐third of cases, there may have been some degree of self‐censorship where opposing views were not raised. Conversely, in approximately one‐fourth (28%) of cases where pre‐discussion ratings were homogeneous, we observed a heterogeneity in voiced views. This suggests that in some cases the discussion context may have prompted participants to consider and articulate alternative viewpoints, despite the homogeneity of their initial ratings.

To investigate variability within heterogeneous groups, we conducted a second chi‐square test of independence comparing the presence of views from both sides of the moral scale between discussions with a 3 versus 1 moral composition (*n* = 150) compared to those with a balanced 2 versus 2 composition (*n* = 174). The percentage of discussions in which views from both sides of the moral scale were voiced is higher when prediscussion ratings were split in a 2 versus 2 composition (74.6%) compared to when they were split in a 3 versus 1 composition (60.1%). The chi‐square test revealed a significant association, (*χ* ^2^(1, *N* = 324) = 6.56, *p* = 0.010), with Cramer's *V* indicating a medium effect size (Cramer's *V* = 0.149). This association suggests that the internal composition of disagreement within heterogeneous groups influenced the likelihood of opposing views being voiced, with more balanced (2 vs. 2) groups fostering a higher likelihood of open moral disagreement compared to asymmetrical (3 vs. 1) ones. This may reflect a reduced social pressure or self‐censorship of viewpoints in more evenly split groups.

### 3.2 Homogeneity Predicts Post‐discussion Extremism

**FIGURE 4:**
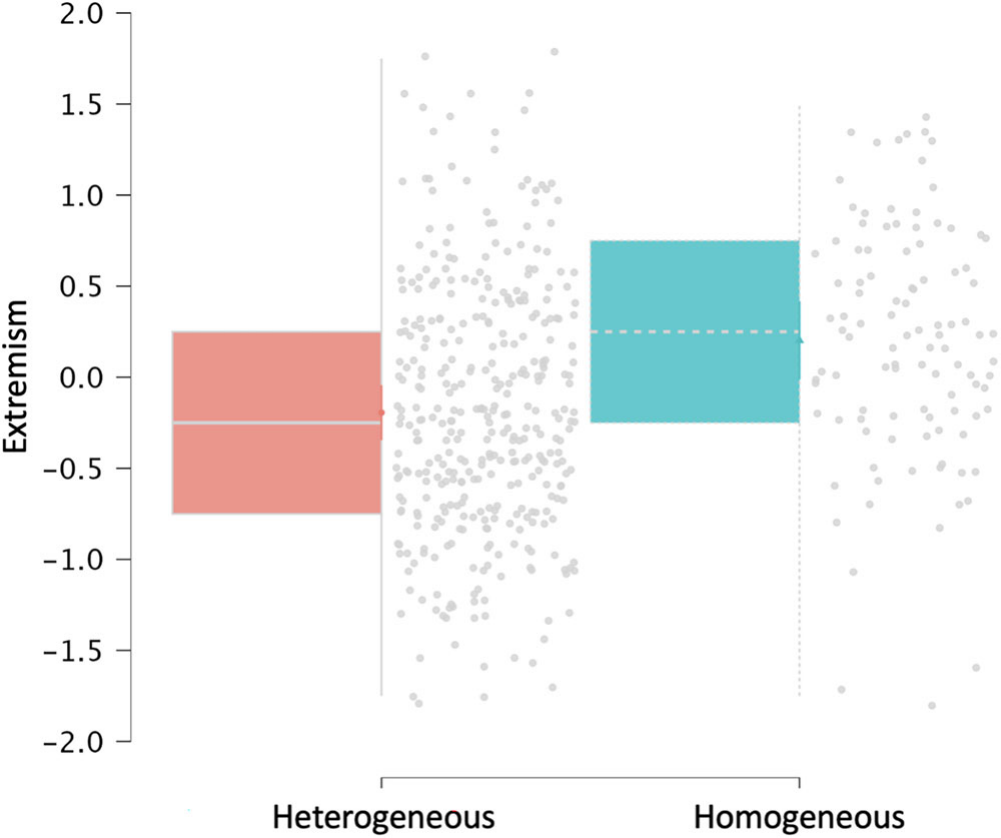
Extremism is higher after discussions in which pre‐discussion ratings were homogeneous. Extremism as a function of whether pre‐discussion ratings were homogeneous or heterogeneous. The gray dots represent individual data points. The colored boxes show the interquartile range between the first and the third quartile. The horizontal line inside each box represents the median. The gray lines extending from the boxes indicate the range of the data. The thick‐colored vertical line shows the 95% confidence interval for the mean.

To examine the relationship between the homogeneity of pre‐discussion opinions and post‐discussion extremism, we employed a random intercepts multilevel model, with extremism as the dependent variable (DV), homogeneity as a dummy‐coded independent variable (IDV), and group and item as crossed random effects. The overall model revealed a significant effect of homogeneity on extremism (*F*_1,464.28_ = 28.36, *p* < 0.001). Specifically, in cases where pre‐discussion ratings were homogeneous, there was a subsequent increase in extremism (*M* = 0.186), whereas in cases where pre‐discussion ratings were heterogeneous, there was a decrease in extremism (*M* = −0.19). The difference between the two conditions was statistically significant (*β*1 = 0.395, SE = 0.074, *t*(464.28) = 5.32, *p* < 0.001; see Figure 4). This indicates that initial homogeneity of opinion is associated with greater subsequent extremism. The variance explained by our model (*R* ^2^) was 0.145, and the effect size (*f* ^2^)—calculated as (*R* ^2^/1 − *R* ^2^)—was 0.169, corresponding a medium effect size [35].

Further, we compared our random intercepts model to a random slopes model but found no significant difference in the models' fit (see Table 1 for a comparison of models). Thus, allowing the slopes to vary by either grouping factor did not improve the model's predictive capability. This suggests that the effect of homogeneity on extremism is consistent across different groups and items (i.e., discussed dilemmas).

**TABLE 1:** Model comparison: Homogeneity predicts post‐discussion extremism

Model	*N* parameters	AIC	BIC	logLik	Chi square	df	*p* value
Null model	4	980.89	997.49	−486.45			
Random intercept multilevel model	5	955.47	976.21	−472.74	27.42	1	< 0.001
Random slopes multilevel model	9	963.34	1000.6	−472.67	0.1303	4	= 0.998

*Note*: This table summarizes the results of comparing the random intercept model (model of interest) to an unconditional means null model and a random slopes model. The comparison is conducted using the ANOVA function in R software.

### 3.3 Interbrain Synchrony Is Higher in Real Compared to Pseudo Groups

To examine differences in interbrain synchrony between real and pseudo groups, we employed a multilevel model. The DV was interbrain synchrony, and group type (real vs. pseudo) was included as the IDV. Item (i.e., the specific dilemma discussed) was treated as a random factor to account for repeated measures across 10 dilemmas per group. Analyses were conducted for each of the 10 brain regions individually, and separately for each experimental condition (discussion and fixation phases), resulting in 20 comparisons.

Across the 20 comparisons, our analysis revealed significant (Bonferroni corrected) differences in synchrony levels between real and pseudo groups for all ROI pairs in the discussion phases. Yet, no difference was detected between the groups in any of the 10 ROI pairs in the fixation phases. Full statistical details for each comparison are presented in Table S1.

These results suggest that levels of interbrain synchrony across all ROI pairs are sensitive to real interaction, as evidenced by the higher levels of synchrony in real groups compared to pseudo groups, and by the fact that this effect was specific to conditions involving interaction, with synchrony differences observed in the discussion phases but not in the fixation phases.

### 3.4 Left DLPFC (BA46) Interbrain Synchrony Mitigates the Effect of Homogeneity on Extremism

We examine the moderating effect of interbrain synchrony on the reported relationship (Section 3.3) between homogeneity and extremism. The analysis employed a random intercept multilevel model (H1 model) with extremism as a DV, homogeneity as a categorical IDV (homogeneous discussions dummy‐coded as 1, heterogeneous discussions dummy‐coded as 0), standardized interbrain synchrony as a continuous moderating IDV (a separate model was run for each ROI pair), and an interaction term between homogeneity and interbrain synchrony. The intercepts were allowed to vary by group and by item (i.e., discussed dilemma). The model allowed us to examine how the relationship between homogeneity and extremism changes at different levels of interbrain synchrony while accounting for potential observation clustering. To assess the fit of our random intercept multilevel model (H1 model), we compared it to a null model with extremism as a DV, homogeneity as a categorical IDV, and where intercepts were allowed to vary by group and by item.

The comparisons between the 10 separate H1 models and their corresponding null models are summarized in Table S2. As observed, out of the 10 ROI pairs, only the H1 model with interbrain synchrony in the left DLPFC (BA46) provided a significantly better fit than the null model (Bonferroni correction was applied to account for the multiple comparisons). The comparison between the null model and the H1 model with interbrain synchrony in the left DLPFC (BA46) is summarized in Table 2. The H1 model demonstrated a significantly better fit than the null model, as indicated by the lower AIC and BIC values, and the higher log‐likelihood. The chi‐square difference test yielded a value of 13.188 with 2 degrees of freedom, which was significant (*p* = 0.0013). The multilevel model's *R* ^2^ value was 0.191, indicating that approximately 19.1% of the variance in extremism levels was explained by the model. The effect size (*f*2 = 0.236) indicates a medium to strong effect [35].

**TABLE 2:** Model comparison: left dlPFC interbrain synchrony moderates the effect of homogeneity on extremism

Model	*N* parameters	AIC	BIC	logLik	Chi square	df	*p* value
Null model	5	904.8	925.16	−447.39			
Random intercept (H1) model	7	895.6	924.13	−440.8	13.188	2	= 0.0013

*Note*: This table summarizes the results of comparing the random intercept model (H1 model) to a null model. The H1 model included homogeneity as a categorical independent variable, standardized interbrain synchrony in the left dorsolateral prefrontal cortex (BA46) as a continuous independent variable, and an interaction term between homogeneity and interbrain synchrony, with random intercepts that vary by group and item. The comparison is conducted using the ANOVA function in R software.

The ANOVA results for the H1 model showed a significant main effect for homogeneity when interbrain synchrony is held constant (*F*_(1, 424.76)_ = 25.147, *p* < 0.001, *β* = 0.386). It further showed a significant interaction effect (*F*_(1, 421.64)_ = 4.553, *p* = 0.033, *β* = −0.17), suggesting that the effect of homogeneity on extremism differs at different levels of interbrain synchrony.

Table 3 reports the results of the simple slopes analysis. Specifically, when standardized interbrain synchrony is at −2.00, the effect of homogeneity on extremism is positive and significant (*β*_1_ = 0.73). As standardized interbrain synchrony increases to −1.00, the effect decreases yet remains positive and significant (*β*_1_ = 0.56). When standardized interbrain synchrony equals 0.00, the effect further decreases but the slope remains significant (*β*_1_ = 0.39). When standardized interbrain synchrony is at 1.00, the slope decreases and turns nonsignificant (*β*_1_ = 0.22). Finally, when standardized interbrain synchrony is at 2.00, the slope further decreases and remains nonsignificant (*β*_1_ = 0.05). These results reveal a significant moderation effect of interbrain synchrony on the relationship between homogeneity and extremism, as the effect of homogeneity on extremism is highest at lower levels of interbrain synchrony, and diminishes as interbrain synchrony increases.

**TABLE 3:** Simple slopes analysis: the effect of homogeneity on extremism at different levels of interbrain synchrony.

IBS's std	Predictor	Estimate	SE	*t*	*p*
−2	Slope	0.73	0.17	4.22	< 0.001
	Conditional intercept	−0.3	0.1	−3.11	0.003
−1	Slope	0.56	0.11	5.2	< 0.001
	Conditional intercept	−0.21	0.08	−2.65	0.017
0	Slope	0.39	0.08	5.01	< 0.001
	Conditional intercept	−0.11	0.07	−1.6	0.138
1	Slope	0.22	0.11	1.89	0.06
	Conditional intercept	−0.02	0.08	−0.25	0.805
2	Slope	0.05	0.18	0.26	0.79
	Conditional intercept	0.07	0.1	0.76	0.450

*Note*: Simple slopes analysis showing the effect of homogeneity on extremism at different levels of interbrain synchrony ranging from −2 standard deviations to 2 in intervals of 1.

Abbreviations: IBS, interbrain synchrony; SE, standard error.

**FIGURE 5:**
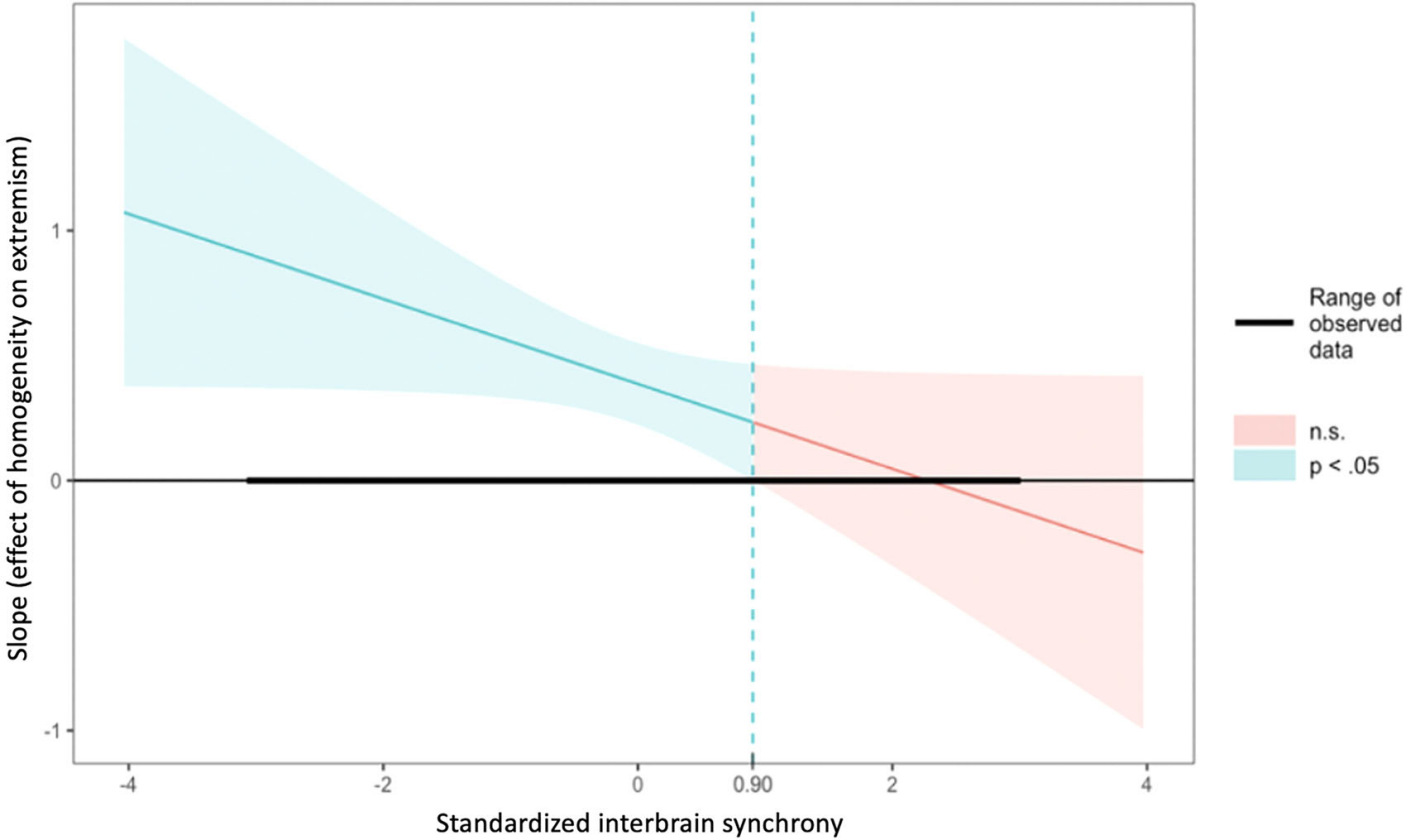
Higher levels of interbrain synchrony in the left dorsolateral prefrontal cortex (DLPFC) diminish the positive effect of homogeneity on extremism. The effect of homogeneity on extremism at different levels of the moderating variable left‐DLPFC interbrain synchrony shown in a Johnson–Neyman plot. The *x*‐axis represents standardized interbrain synchrony values, which empirically range from −3.05 to 2.98 SD as indicated by the black horizontal line on the plot, and the *y*‐axis represents the effect of homogeneity on extremism (slope estimate). The solid colored line depicts the estimated effect, while the surrounding shaded color area represents the 95% confidence intervals and represents regions where the effect is significant (blue) or nonsignificant (red). The plot indicates that the effect of homogeneity on extremism is statistically significant when interbrain synchrony values are below 0.90 (left side of vertical dashed line), and nonsignificant above it. Within the significant range, the effect is positive, meaning that as homogeneity increases, so does extremism. Yet, above 0.90 the effect is not significantly different from zero indicating no relationship between homogeneity and extremism within this range.

To specify the range of interbrain synchrony values where the effect of homogeneity on extremism is significant, we applied the Johnson–Neyman technique at a significance level of 0.05 (FDR corrected). The Johnson–Neyman plot is shown in Figure 5. The observed range of standardized interbrain synchrony values in the data was between −3.05 and 2.98. This analysis indicated that the positive effect of homogeneity on extremism is statistically significant when standardized interbrain synchrony is between −3.05 and 0.90 standard deviations. For the range of standardized interbrain synchrony values above the 0.90 threshold, the effect was no longer significant. This indicates that when interbrain synchrony in the left DLPFC is high, particularly above 0.90 standard deviations, the association between homogeneity and extremism is diminished such that there is no difference in extremism between homogeneous and heterogeneous groups. However, when interbrain synchrony is low, the association between homogeneity and extremism is stronger, with homogeneity predicting higher extremism. These findings suggest that higher levels of interbrain synchrony in the left DLPFC mitigate the echo chamber effect, where homogeneity increases extremism.

Lastly, we were further interested in exploring putative predictors of DLPFC interbrain synchrony to better understand what group‐level factors might shape its variability across different groups. In particular, we examined whether group dominance structure impacted average IBS in the left DLPFC. Group dominance structure (see Supporting Information Section S1.1) captures the dominance level similarity between group members by calculating the variance of dominance scores between group members, such that a group with zero variance would be a completely uniform group where all members have the same degree of dominance, and a group with higher variance would be considered less uniform. A linear regression analysis revealed that dominance variance did not significantly predict group‐level IBS (*β* = 0.0007, SE = 0.002, *t*(44) = 0.30, *p* = 0.765, *R* ^2^ = 0.002). These findings suggest that differences in dominance structure do not account for the variability in interbrain synchrony observed across groups.

## 4 Discussion

The study of interbrain dynamics during group discussions offers a direct lens into the mechanisms of information exchange between individuals, which can shape cultural shifts while posing potential risks for extremism. The present study explored how discussions within homogeneous groups drive extremism and examined whether interbrain synchrony—in regions related to executive functions—between group members can moderate this fueling effect of homogeneity on extremism.

The first key finding demonstrates that groups with homogeneous opinions are significantly more likely to form echo chamber‐like environments, where views supporting only one side of the normative moral spectrum are voiced. This finding aligns with prior research showing that pre‐existing homogeneity restricts the range of discussed perspectives within a group [36]. Interestingly, within homogeneous groups, we did not observe complete correspondence between homogeneity in pre‐discussion ratings and homogeneity in voiced opinions during the discussion. Additionally, within heterogeneous groups, we observed that balanced groups, in which two group members had a similar moral inclination that is different from the one shared by the other two group members, exhibited a higher likelihood of voicing opposing views compared to asymmetrical (3 vs. 1) groups. These results may reflect a reduced social pressure or self‐censorship of viewpoints in more evenly split groups, and suggest that discussions have the potential to either suppress alternative perspectives or introduce new ones, likely depending on the dynamics within the group and the context of the discussion [5].

Another goal of the current study was to test whether we can generate an echo chamber effect by demonstrating that discussions within homogeneous groups drive extremism post‐discussion. The results strongly support the hypothesis that pre‐discussion homogeneity in opinions predicts higher levels of post‐discussion extremism. Homogeneous groups exhibited increased extremism, while heterogeneous groups experienced a decrease, suggesting that diversity in viewpoints serves as a buffer against shifts toward extreme positions. This finding aligns with existing theories on group extremism, which suggest that exposure to divergent viewpoints can moderate individual opinions [37, 38, 39].

Lastly, the main goal of the current study was to explore the neural mechanisms that may mitigate the echo chamber effect in homogeneous groups. Specifically, we examined whether interbrain synchrony could moderate the impact of homogeneity on extremism. Our findings show that the effect of homogeneity on extremism was moderated by interbrain synchrony in the left DLPFC (BA 46) between group members. Specifically, higher interbrain synchrony was associated with a reduction in the effect of homogeneity on extremism. Notably, this effect was not influenced by group‐level dominance structure, which did not significantly predict interbrain synchrony.

Our findings align with and extend prior research on the neural underpinnings of political and ideological extremism. Recent work demonstrated that intolerance of uncertainty modulates interbrain synchrony—in regions related to valuation and affect—between ideologically homogeneous individuals exposed to politically polarized content [40]. While their focus was on perceptual alignment during shared media exposure in the absence of social interactions, our study builds on this foundation by examining interbrain synchrony—in a region related to executive functions—in the context of live, interactive group discussions. This shift toward naturalistic, face‐to‐face communication enabled us to investigate how different patterns of interbrain synchrony can not only reflect shared perception but may also serve as a mechanism for mitigating attitudinal extremity through social deliberation.

Our interbrain synchrony findings are also consistent with prior studies showing that patients with DLPFC lesions are more prone to dogmatism and fundamentalist thinking [23]. One mechanism that may explain the relationship between reduced DLPFC activity and extremism is the role this region plays in supporting cognitive flexibility. The DLPFC is critical for adaptive shifting of behavior and the inhibition of automatic, rigid responses [41]. Crucially, this cognitive flexibility has been shown to inversely relate to ideological extremity: in a large behavioral study, researchers found that partisan extremity was associated with lower levels of cognitive flexibility, regardless of political orientation [42]. These findings suggest that DLPFC interbrain synchrony, through its support of flexible information processing, may play a critical role in reducing emergent extremism by helping individuals—and groups—entertain multiple perspectives and revise beliefs in light of new evidence. Indeed, previous research implicates left DLPFC interbrain synchrony in a wide range of group tasks that require co‐mental flexibility, such as cooperation and joint creativity tasks [14, 43].

While our findings demonstrate a statistical moderation effect, we caution against interpreting this as evidence of a direct causal influence of interbrain synchrony. Instead, we conceptualize synchrony as a relational state that either facilitates or reflects an emergent group‐level mindset under which homogeneity leads to more or less extreme outcomes. We propose that this flexible mindset can buffer extremism by offsetting the cognitive‐motivational mechanisms that underlie echo chambers [44]. For example, within echo chambers, the selective exposure bias can limit the tendency to question shared beliefs due to members’ desire to confirm those beliefs and to maintain alignment within the group [45, 46, 47]. A flexible mindset shared among group members, may reduce the impact of selective exposure bias by embracing decision uncertainty and increasing openness to alternative perspectives that might challenge pre‐existing group beliefs [44, 48]. Similarly, availability bias—relying on the most easily retrieved or commonly voiced information [49]—can lead to overconfidence in dominant group opinions [50]. A flexible group mindset can counter this tendency by fostering a more balanced and nuanced evaluation of alternatives. These proposals require further empirical investigation to unpack the causal pathways linking interbrain synchrony, cognitive flexibility, and extremism.

Our study advances the literature on echo chambers in two key ways. First, we propose a novel paradigm for examining the echo chamber effect in a laboratory context using face‐to‐face group interactions. This stands in contrast to prior research, which has predominantly relied on computational simulations of opinion cascades or models of echo chamber dynamics within social media environments [51, 52]. While these approaches have yielded important insights, they often struggle to disentangle causality due to the self‐selective nature of online communities. In such environments, homogeneity and extremism may both arise from underlying tendencies to affiliate with like‐minded individuals and conform to shared views—making it difficult to isolate the causal role of group composition [51, 52, 53]. This potential confound could inflate the supposed prevalence of echo chambers [54]. By studying randomly assembled groups in controlled, naturalistic settings, our paradigm enables stronger causal inference and offers a clearer lens into the social mechanisms underpinning extremism [55]. Second, our approach offers an interactive group paradigm that integrates behavioral and neuroscientific methods to offer the first investigation of the naturally emerging neural mechanisms that differentiate homogeneous groups that display an echo chamber effect from those that do not. Our understanding of extremism remains limited in part because core processes such as selective exposure and social conformity are difficult to reproduce in static, decontextualized laboratory settings [59]. By moving beyond isolated settings and focusing on interbrain dynamics during live interactions, our approach offers a more ecologically valid framework for understanding how social context and neural mechanisms jointly shape polarized attitudes. This approach opens new avenues for identifying both the drivers of the echo chamber effect and potential mechanisms for mitigating it.

Our results have significant implications for research on reducing extremism. Previous approaches to reducing the echo chamber effect focused on dismantling ideological homogeneity, either by nudging individuals to encounter diverse perspectives or by introducing impartial discussion facilitators [9, 56, 57]. However, these approaches may be insufficient in the long term, given our innate tendency toward homophily, which reinforces the attraction to homogeneity [46, 58]. Our results suggest an alternative strategy: promoting group states that can act as an antidote to the echo chamber effect, rather than focusing on reducing homogeneity itself.

Several limitations of our study warrant consideration. First, the groups were randomly assembled, which may have attenuated factors that typically drive extremism in echo chambers, such as strong group identity or ideological alignment. Second, participants engaged in discussions about abstract moral dilemmas, which may not fully reflect the dynamics of real‐world conversations, particularly those centered on emotionally charged or personally relevant topics. Finally, while our study examined how contextual group features (e.g., homogeneity) and interpersonal phenomena (e.g., interbrain synchrony) influence extremism, we did not assess individual cognitive traits—such as intolerance of uncertainty, need for closure, or cognitive rigidity—that may interact with these contextual factors [59]. As Van Baar and FeldmanHall [59] suggest, investigating how such traits interact with social and neural dynamics may be key to understanding the emergence and variability of extremism. Future research should incorporate assessments of individual cognitive traits to explore how personal and contextual factors jointly shape susceptibility to extremism in echo chamber environments. While our study provides insights into one potential mechanism for mitigating the echo chamber effect, this mechanism alone is unlikely to be sufficient in addressing the complexities of emergent extremism in real‐world social contexts.

## 5 Conclusion

In conclusion, our study contributes to the growing body of literature on the echo chamber effect and identifies potential strategies for mitigating extremism. These findings provide valuable insights into addressing societal challenges such as ideological divides, political gridlock, and sectarianism. Crucially, our research highlights the importance of integrating behavioral and neuroscientific approaches to deepen our understanding of the mechanisms underlying social phenomena.

## Author Contributions

The authors confirm contribution to the paper as follows: Conceptualization: Aial Sobeh, Tomer Marcos Vakrat, and Simone Shamay‐Tsoory; Methodology: Aial Sobeh, Tomer Marcos Vakrat, and Simone Shamay‐Tsoory; Investigation: Aial Sobeh and Tomer Marcos Vakrat; Formal Analysis: Aial Sobeh and Simone Shamay‐Tsoory; writing—original draft preparation: Aial Sobeh, Tomer Marcos Vakrat, and Simone Shamay‐Tsoory. All the authors reviewed the results and approved the final version of the manuscript.

## Conflicts of Interest

The authors declare no conflicts of interest.

## Supporting information




**Supplementary Materials**: nyas70083‐sup‐0001‐SuppMat.docx

## Data Availability

The data that support the findings of this study are openly available in Mendeley Data at https://doi.org/10.17632/6rhg32kf7g.1.
