# Supplementary material for: Interbrain Synchrony Mitigates Extremism Within Echo Chambers
Source: Ann N Y Acad Sci. 2025 Sep 12;1552(1):117–28. doi: 10.1111/nyas.70083 (PMC12576876; doi:10.1111/nyas.70083)
Supplement: Supplementary file 1 — Supplementary Materials: nyas70083‐sup‐0001‐SuppMat.docx [file NYAS-1552-117-s001.docx]

**Interbrain Synchrony Mitigates Extremism Within Echo Chambers**

**Supporting Information**

**S1. Supporting Methods.**

**S1.1. Group Dominance Structure**

To capture the dominance dynamics within each group, we relied on external evaluations provided by independent raters. Specifically, two trained research assistants observed each of the ten deliberation sessions and, following each phase, independently identified the group member they perceived as the most dominant. This process yielded two independent judgments per session, resulting in a total of twenty dominance judgments per group across all sessions.

Each time a participant was identified as the most dominant by a rater, they received one point. These points were aggregated across all sessions, producing an individual dominance score ranging from 0 (never selected) to 20 (consistently selected by both raters). Since each group consisted of four members, the total of 20 points was distributed among them based on perceived dominance.

To quantify the overall dominance structure of the group, we computed the variance of these dominance scores. A low variance indicates a relatively equal distribution of influence (i.e., an egalitarian structure), while a high variance reflects greater asymmetry in perceived dominance, signifying a more centralized structure.

**Supporting Figure S1.**

*A moral dilemma and the moral appropriateness scale*


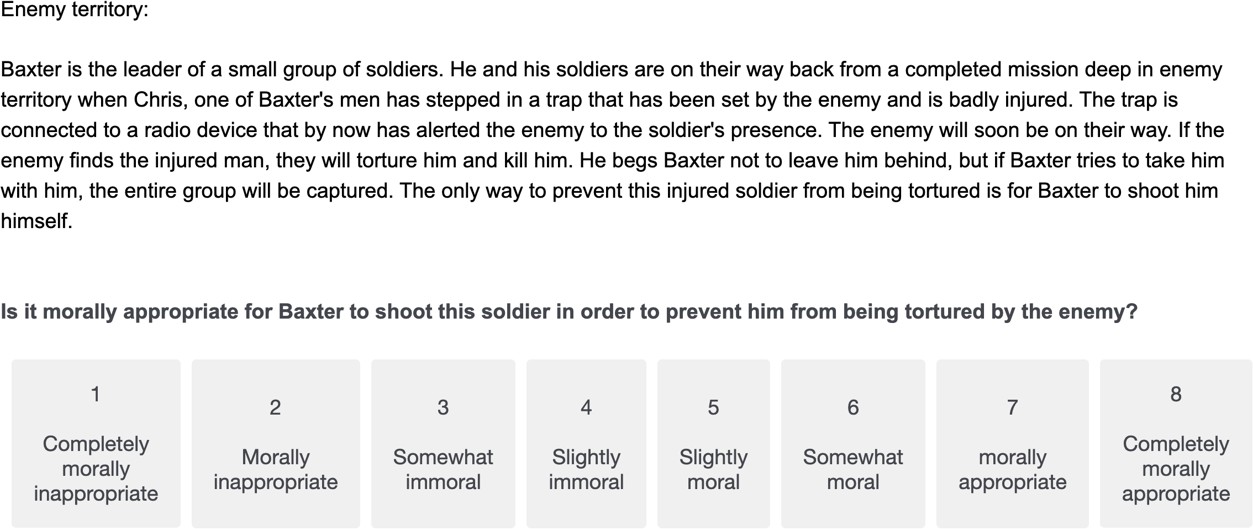


*Note.* A screenshot of a computer screen in which one dilemma (out of ten) is presented to the participants in our study. Underneath is the interactive moral appropriateness scale. Note that the content in the screen is translated here to English, whereas the actual content in our study was in either Hebrew or Arabic.

**Supporting Table S1**

*Comparing Interbrain synchrony levels between real and pseudo groups across all ROI pairs and across both experimental conditions.*

| **Condition** | **ROI pair** | **β_1_** | **SE** | **DF** | ***T* value** | ***P* Value** | **bonferroni corrected sig.** |
| --- | --- | --- | --- | --- | --- | --- | --- |
| Discussion | Left dlPFC (BA46) | 0.014 | 0.0032 | 877.31 | 4.3822 | < .001 | TRUE |
| Discussion | Right dlPFC (BA46) | 0.0106 | 0.0027 | 896 | 3.8308 | < .001 | TRUE |
| Discussion | Left dlPFC (BA9) | 0.0138 | 0.0029 | 887.09 | 4.7806 | < .001 | TRUE |
| Discussion | Right dlPFC (BA9) | 0.0125 | 0.0027 | 883.79 | 4.5388 | < .001 | TRUE |
| Discussion | Left pars opercularis | 0.0126 | 0.0028 | 897 | 4.3856 | < .001 | TRUE |
| Discussion | Right pars opercularis | 0.0114 | 0.0027 | 896 | 4.1522 | < .001 | TRUE |
| Discussion | Left pars triangularis | 0.0109 | 0.0027 | 887.56 | 4.0225 | < .001 | TRUE |
| Discussion | Right pars triangularis | 0.0163 | 0.0028 | 887.62 | 5.7698 | < .001 | TRUE |
| Discussion | Left premotor cortex | 0.0104 | 0.0028 | 887.74 | 3.7337 | < .001 | TRUE |
| Discussion | Right premotor cortex | 0.0133 | 0.0029 | 886.41 | 4.6044 | < .001 | TRUE |
| Fixation | Left dlPFC (BA46) | 0.0062 | 0.0044 | 876.6 | 1.3973 | 0.1626 | FALSE |
| Fixation | Right dlPFC (BA46) | 0.0054 | 0.0040 | 885.53 | 1.3373 | 0.1814 | FALSE |
| Fixation | Left dlPFC (BA9) | 0.0085 | 0.0041 | 886.4 | 2.0659 | 0.0391 | FALSE |
| Fixation | Right dlPFC (BA9) | 0.0067 | 0.0039 | 885.29 | 1.6984 | 0.0897 | FALSE |
| Fixation | Left pars opercularis | 0.0071 | 0.0039 | 886.56 | 1.791 | 0.0736 | FALSE |
| Fixation | Right pars opercularis | 0.009 | 0.0042 | 885.5 | 2.1517 | 0.0316 | FALSE |
| Fixation | Left pars triangularis | 0.0107 | 0.0040 | 886.37 | 2.6735 | 0.0076 | FALSE |
| Fixation | Right pars triangularis | 0.0103 | 0.004192 | 886.42 | 2.4659 | 0.0138 | FALSE |
| Fixation | Left premotor cortex | 0.01 | 0.0043 | 886.42 | 2.3165 | 0.0207 | FALSE |
| Fixation | Right premotor cortex | 0.0051 | 0.0042 | 885.25 | 1.2041 | 0.2288 | FALSE |

*Note.* This table summarizes the results of the twenty combarsions comparing interbrain synchrony value in real groups (dummy coded as 1) to pseudo groups (dummy coded as 0), across various ROI pairs and in both experimental conditions. That table includes the estimate of beta (synchrony in real groups minus synchrony in pseudo groups), standard error (SE), degrees of freedom (DF), t-value, p-value, and whether or not the p-value is significant compared to a Bonferroni corrected critical p.

**Supporting Table S2**

*Model comparisons between the ten separate H1 models and their corresponding null models.*

| **ROI pair** | **Model** | **N paramters** | **AIC** | **BIC** | **Chi square** | **DF** | **p value** |
| --- | --- | --- | --- | --- | --- | --- | --- |
| Left dlPFC (BA46) | Null Model | 5 | 904.785 | 925.162 |  |  |  |
|  | H1 Model | 7 | 895.597 | 924.125 | 13.187 | 2 | 0.0013 |
| Right dlPFC (BA46) | Null Model | 5 | 927.1426 | 947.6217 |  |  |  |
|  | H1 Model | 7 | 929.080 | 957.751 | 2.0619 | 2 | 0.3566 |
| Left dlPFC (BA9) | Null Model | 5 | 923.408 | 943.876 |  |  |  |
|  | H1 Model | 7 | 926.684 | 955.339 | 0.7242 | 2 | 0.6961 |
| Right dlPFC (BA9) | Null Model | 5 | 919.187 | 939.643 |  |  |  |
|  | H1 Model | 7 | 921.541 | 950.180 | 1.6461 | 2 | 0.439 |
| Left pars opercularis | Null Model | 5 | 928.323 | 948.814 |  |  |  |
|  | H1 Model | 7 | 932.202 | 960.889 | 0.121 | 2 | 0.9412 |
| Right pars opercularis | Null Model | 5 | 926.143 | 946.622 |  |  |  |
|  | H1 Model | 7 | 929.962 | 958.633 | 0.1808 | 2 | 0.9135 |
| Left pars triangularis | Null Model | 5 | 926.396 | 946.875 |  |  |  |
|  | H1 Model | 7 | 929.047 | 957.718 | 1.3491 | 2 | 0.5093 |
| Right pars triangularis | Null Model | 5 | 922.767 | 943.224 |  |  |  |
|  | H1 Model | 7 | 925.579 | 954.218 | 1.1883 | 2 | 0.552 |
| Left premotor cortex | Null Model | 5 | 925.905 | 946.3729 |  |  |  |
|  | H1 Model | 7 | 925.882 | 954.537 | 4.0227 | 2 | 0.1338 |
| Right premotor cortex | Null Model | 5 | 916.065 | 936.499 |  |  |  |
|  | H1 Model | 7 | 917.485 | 946.092 | 2.5807 | 2 | 0.2751 |

*Note.* This table summarizes the results of the ten model comparisons.
